# First Detection of Mukawa Virus in *Ixodes persulcatus* and *Haemaphysalis concinna* in China

**DOI:** 10.3389/fmicb.2022.791563

**Published:** 2022-03-03

**Authors:** Yu-Na Wang, Rui-Ruo Jiang, Heng Ding, Xiao-Long Zhang, Ning Wang, Yun-Fa Zhang, Yue Li, Jin-Jin Chen, Pan-He Zhang, Hao Li, Jia-Fu Jiang, Lan-Zheng Liu, Meng-bin Yu, Gang Wang, Xiao-Ai Zhang, Wei Liu

**Affiliations:** ^1^State Key Laboratory of Pathogen and Biosecurity, Beijing Institute of Microbiology and Epidemiology, Beijing, China; ^2^Institute of NBC Defence, PLA Army, Beijing, China; ^3^Science and Technology Research Center of China Customs, Beijing, China; ^4^Shandong Center for Disease Control and Prevention, Jinan, China

**Keywords:** ticks, tick-borne phlebovirus, *Ixodes persulcatus*, China, *Haemaphysalis concinna*

## Abstract

Mukawa virus (MKWV), a novel tick-borne virus (TBV) of the genus *Phlebovirus* of family *Phenuiviridae*, has been firstly reported in *Ixodes persulcatus* in Japan. In this study, we made an epidemiological investigation in China to obtain the geographic distribution and genetic features of this virus outside Japan. We screened 1,815 adult ticks (665 *I. persulcatus*, 336 *Dermacentor silvarum*, 599 *Haemaphysalis longicornis*, 170 *Rhipicephalus microplus*, 45 *Haemaphysalis concinna*) and 805 wild small mammals collected from eight provinces. The positive rate of 6.77% (45/665, including 18 female and 27 male *I. persulcatus*) and 2.22% (1/45, 1 male *H. concinna*) were obtained from *I. persulcatus* and *H. concinna* in Heilongjiang province, respectively. No evidence of MKWV infection was found in other three tick species or any of the mammalian species. The virus can infect the Vero cells successfully, indicating the ability of MKWV to replicate in mammalian cells. A phylogenetic tree based on the nucleotide sequences of L, M, and S segments demonstrated that the Japanese MKWV variant, our two MKWV variants, and KURV were clustered with the members of the mosquito/sandfly-borne phleboviruses and distant from other tick-borne phenuiviruses. A phylogenetic analysis based on 895 bp partial L gene sequences (*n* = 46) showed that all MKWV sequences were separated into three lineages. Our results showed the presence of MKWV in *I. persulcatus* and *H. concinna* in northeast of China, highlighting the necessity of epidemiological study in wider regions. Due to the ability of MKWV to replicate in mammalian cells, the potential for zoonosis, and wide distribution of *I. persulcatus* and *H. concinna* in China, the important vectors of MKWV, further screening to more tick species, wild animals, domestic animals, and humans raises up practical significance.

## Introduction

In the past several years, the scope and variety of the tick-borne infectious disease (TBD) have increased dramatically, driven largely by the use of molecular diagnostic techniques that have facilitated the identification of novel tick-borne viruses (TBVs). The most well-known example is the discovery of two novel TBVs in the family *Phenuiviridae* associated with severe disease and death in human cases in Asia and the United States, i.e., severe fever with thrombocytopenia syndrome virus (SFTSV) and Heartland virus (HRTV; Yu et al., 2011; McMullan et al., 2012). SFTSV was firstly discovered as life-threatening novel bunyavirus in China in 2009; the taxonomical species designation has recently been renamed *Dabie bandavirus*, classified in the genus *Bandavirus*, family *Phenuiviridae* in the order *Bunyavirales* (Casel et al., 2021). SFTSV infections have been also reported in humans in South Korea, Japan, Vietnam, and Pakistan (Kim et al., 2013; Takahashi et al., 2014; Tran et al., 2019; Zohaib et al., 2020). HRTV was discovered in the United States in 2011, most closely related to but clearly distinct from SFTSV (McMullan et al., 2012). In 2018, another novel TBV that was also grouped into the genus *Phlebovirus* of the family *Phenuiviridae* called Mukawa virus (MKWV), was isolated from host-questing *Ixodes persulcatus* in Hokkaido, Japan (Matsuno et al., 2018; Abudurexiti et al., 2019). Despite its genetic similarity to mosquito/sandfly-borne phleboviruses, the molecular footprints of viral proteins and biological characteristics defined MKWV as more like a tick-borne phlebovirus (TBPV; Matsuno et al., 2018). Soon after its discovery, a serological survey in wildlife disclosed the presence of neutralizing antibodies against MKWV in both Yezo deer and raccoons captured in the first-discovery place in Hokkaido (Torii et al., 2019). This finding supported the establishment of endemic foci for MKWV with zoonotic potential in Japan. Except for the epidemiology evidence from Japan, no distribution in other countries or information regarding the molecular evolution of MKWV was available. Considering the wide geographic distribution of tick-borne phenuiviruses other than MKWV in Eastern Asia (China, Japan, South Korea), it is tempting to hypothesize that MKWV may also exist in tick populations in China. In this study, we detected various species of small mammals and dominant tick species in eight provinces across China, determining the presence of MKWV in *I. persulcatus* and *H. concinna* in northeastern China. This knowledge might help to improve our understanding of the epidemiological feature of MKWV, to guide diagnostic and treatment algorithms in case of causing human infection in the future.

## Materials and Methods

### Study Site and Sample Collection

From September 2017 to October 2021, free-living or engorged ticks were collected by dragging a frag, and wild small mammals were captured by snap traps from eight provinces (Heilongjiang, Xinjiang, Inner Mongolia, Henan, Shanxi, Liaoning, Shandong, and Zhejiang) in China. Tick species were identified based on morphological characteristics, while the wild small mammals were identified by morphological features to the species level and further confirmed by the sequencing of the mitochondrial cytochrome *b* (*mt-cyt b*) gene (Nicolas et al., 2006). Organ samples including heart, lung, liver, kidney, and spleen were obtained from each wild small mammal in the BSL-2 laboratory. All collected samples were stored in tubes at −80°C prior to use.

### Extraction of Viral RNA and One-Step Reverse Transcription-Polymerase Chain Reaction

All collected ticks were thoroughly surface-sterilized with 70% ethanol, followed by distilled sterile water, and then each individual tick and the aliquot of each organ of wild small mammals were homogenized by using small steel balls as an abrasive. Total viral nucleic acids were extracted by using All Prep DNA/RNA Mini Kit (Qiagen, Hilden, Germany) according to the manufacturer’s protocol. A one-step reverse transcription-polymerase chain reaction (RT-PCR) system based on two primer sets (HRTV and TBPV) that were universal for the detection of TBPV was applied to test the extracted RNAs according to a previous report (Table 1; Matsuno et al., 2015). PCR amplification was performed using the PrimeScript one-step RT-PCR kit version 2 (TaKaRa) following the manufacturer’s instructions, under the following program: 50°C for 30 min; 94°C for 2 min; 40 cycles of 94°C for 30 s, 55°C for 30 s, and 72°C for 30 s; and 72°C for 5 min. The amplified products were detected by agarose gel electrophoresis to confirm their sizes and then subjected to Sanger sequencing.

**TABLE 1:** Tick and small wild mammals screened for MKWV.

Tick and small wild mammals	Location	Year	Species (n)	No. total tested	No. (%) of MKWV positive samples
Tick	Heilongjiang	2019, 2021	*Ixodes persulcatus* (665), *Haemaphysalis concinna* (45)	710	46 (6.48)
	Inner Mongolia	2019	*Dermacentor silvarum* (336)	336	0 (0)
	Henan	2019	*Haemaphysalis longicornis* (109)	109	0 (0)
	Shanxi	2019	*Haemaphysalis longicornis* (144)	144	0 (0)
	Liaoning	2019	*Haemaphysalis longicornis* (174)	174	0 (0)
	Shandong	2019	*Haemaphysalis longicornis* (172)	172	0 (0)
	Zhejiang	2018	*Rhipicephalus microplus* (170)	170	0 (0)
	Total			1815	46 (2.53)
Small wild mammals	Heilongjiang	2017	*Reed Vole* (5), *Brown Rat* (22), *Striped Field Mouse* (16), *Siberian Chipmunk* (1), *House Mouse* (1)	45	0 (0)
	Inner Mongolia	2019	*Daurian Ground Squirrel* (35), *Eversman’s Hamster* (4), *Striped Dwarf Hamster* (2), *Gray Hamster* (38), *Djungarian Hamster* (6), *Northern Three-toed Jerboa* (5), *Mongolian five-toed Jerboa* (28), *House Mouse* (2), *Desert Hamster* (4), *Mongolian Gerbil* (183), *Mid-day Gerbil* (64), *Microtus mandarinus* (15)	386	0 (0)
	Henan	2018	*Striped Field Mouse* (15), *Confucian Niviventer* (1), *Bower’s White-toothed Rat* (2), *Brown Rat* (5)	23	0 (0)
	Xinjiang	2018	*Tamarisk Gerbil* (3), *Red-cheeked Ground Squirrel* (31), *Yunnan Red-backed Vole* (1), *Great Gerbil* (77), *Müller’s giant Sunda Rat* (4), *Root Vole* (1), *Brown Rat* (20), *Libyan Jird* (48), *Marbled Polecat* (1), *Gray Hamster* (5), *Least Weasel* (1), *shrew* (2), *Taiwan vole* (1), *Mongolian five-toed Jerboa* (8), *Himalayan Marmot* (1), *House Mouse* (104), *Field Mouse* (15), *Arctic Ground Squirrel* (25), *Mid-day Gerbil* (3)	351	0 (0)
	Total			805	0 (0)

Primers (5′-CCATCAATCTGTACACCAGG-3′ and 5′- ACACAAAGTCCGCCCATTAC-3′) targeting the 895 bp fragment of the large (L) gene were used to confirm the positive results (Table 2). PCR amplification was performed using the PrimeScript one-step RT-PCR kit version 2 (TaKaRa) following the manufacturer’s instructions, under the following program: initial denaturation: 50°C for 30 min; 94°C for 2 min; 13 cycles, decreasing the annealing temperature 0.5°C each cycle: 94°C, 30 s; 57°C, 30 s (0.5°C/cycle); 72°C, 1 min; 37 cycles: 94°C for 30 s, 51°C for 30 s, and 72°C for 1 min; and 72°C for 5 min. The amplified products were detected by agarose gel electrophoresis and then subjected to Sanger sequencing. All positive and negative results were reconfirmed by real-time RT-PCR with the MKWV-specific primers (L-6314-6443F, L-6314-6443R) (Table 1). All PCR tests were conducted in parallel with positive control (RNA from positive sample) and negative control (RNase-free water).

**TABLE 2:** Primers used in the RT-PCR for MKWV detection.

Primer set	Primer	Sequence (5′→3′)	Methods
HRTV	HRT-GL2759F	CAGCATGGIGGIYTIAGRGAAATYTATGT	RT-PCR based sequencing
	HRT-GL3276R	GAWGTRWARTGCAGGATICCYTGCATCAT	
TBPV	TBPVL2759F	CAGCATGGIGGICTIAGAGAGAT	RT-PCR based sequencing
	TBPVL3267R	TGIAGIATSCCYTGCATCAT	
L-5549-6443	L-5549-6443F	CCATCAATCTGTACACCAGG	RT-PCR based sequencing
	L-5549-6443R	ACACAAAGTCCGCCCATTAC	
L-6314-6443	L-6314-6443F	AGAGCTTGCCATGAAACAG	Real-time RT-PCR
	L-6314-6443R	ACACAAAGTCCGCCCATTAC	

### Virus Isolation

Positive ticks were used for MKWV isolation. Briefly, the homogenate of positive ticks were filtered and inoculated into pre-cultured Vero, BHK, and Huh-7 cells in dulbecco’s modified eagle medium (DMEM) medium supplemented with 10% fetal bovine serum (FBS), 1% penicillin–streptomycin (GIBCO) that were maintained under 5% CO_2_ at 37°C. One hour after inoculation, the medium was replaced and then cells were further cultured at 37°C for three generations (each cultured for 7 days). During this period, cytopathic effects (CPEs) were observed daily and MKWV-specific RNA was tested on each generation equalization from the supernatant by the same RT-PCR method as that used for the sample detection mentioned above.

### Determination of Full-Length Viral Nucleotide Sequences

The full-length genome sequence of MKWV was obtained by sequencing the MKWV-positive cell supernatant and MKWV-positive ticks. Briefly, RNA was extracted from the cell supernatant, and three fragments of MKWV were amplified by specific primers (Supplementary Table 1) designed according to the submitted nucleotide sequences (accession numbers LC063768-LC063770). The 5′ and 3′ termini sequences of each segment were determined with a rapid amplification of cDNA ends Kit (Invitrogen, Waltham, MA, United States). The purified PCR products were directly sequenced to determine the complete genome sequence of MKWV.

### Phylogenetic Analysis

The nucleotide and amino acid sequences from the currently detected MKWV and other representative species from family *Phenuiviridae* that were downloaded from GenBank were aligned by the ClustalW method using MEGA-X. Phylogenetic trees were constructed by using Hainan oriental leaf-toed gecko hantavirus in the genus *Reptillovirus* of the family *Hantaviridae* as an outgroup. The amino acid sequences of nucleocapsid proteins (N) and non-structural proteins (NSs) were further aligned by ClustalW. Phylogenetic trees were constructed using the maximum likelihood (ML) method and the robustness of each node was tested by 1,000 bootstrap replications.

## Results

### Detection of Mukawa Virus in Ticks and Wild Small Mammals

**FIGURE 1:**
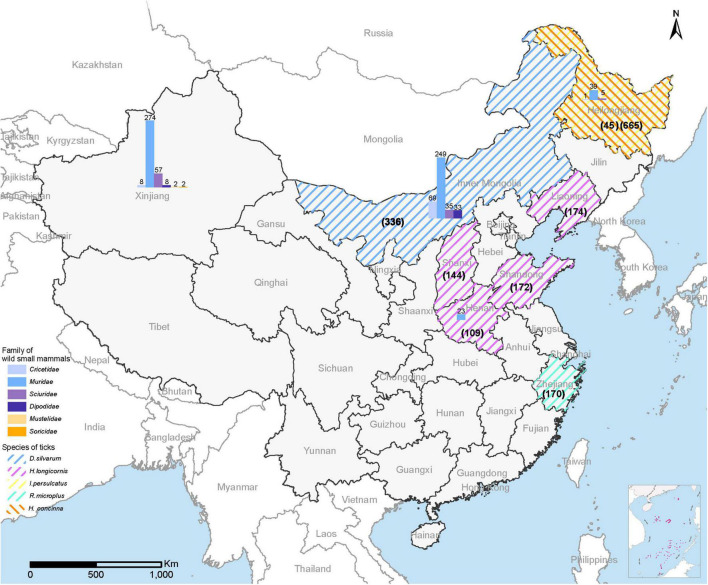
Map of China showing the collection sites for ticks and wild small mammals for MKWV detection. The sampling number for each tick was shown in parentheses; the sampling number for each animal species was marked above the column.

From September 2017 to October 2021, a total of 1,815 adult ticks (665 *I. persulcatus*, 336 *Dermacentor silvarum*, 599 *Haemaphysalis longicornis*, 170 *Rhipicephalus microplus*, and 45 *H. concinna*) and 805 wild small mammals were collected in eight provinces (Heilongjiang, Inner Mongolia, Xinjiang, Shandong, Henan, Shanxi, Liaoning, and Zhejiang) in China (Figure 1). The positive detection for MKWV was determined from 2.5% (46/1,815) of ticks, including *I. persulcatus* (6.77%, 45/665) and *H. concinna* (2.22%, 1/45), both significantly higher than those obtained from other tick species (0% for 336 *D. silvarum*, 599 *H. longicornis*, and 170 *R. microplus)* (Table 1). Significantly higher positive detection was observed from male than from female *I. persulcatus* and *H. concinna* ticks (60 vs. 40%, 67 vs. 33%). All the positive results were obtained from ticks captured in Heilongjiang province.

A total of 805 wild small mammals belonging to 37 species in the *Cricetidae*, *Dipodidae*, *Muridae*, and *Sciuridae* family of *Rodentia*, 1 species in the *Soricidae* family of *Soricomorpha*, 2 species in the *Mustelidae* family of *Carnivora* from four provinces (Heilongjiang, Henan, Xinjiang, Inner Mongolia) were examined. No positive detection for MKWV was obtained for each organ (Table 1).

### Isolation and Genome Characterization of Mukawa Virus

Among three inoculated cultured cells (Vero, BHK, and Huh7 cells), only Vero cells showed CPE on the third and subsequent serial passages, which were confirmed to be positive for MKWV by performing RT-PCR on the RNA extracted from the supernatant of culture. No CPE or positive MKWV RNA detection was obtained in the inoculated BHK and Huh7 cells.

**FIGURE 2:**
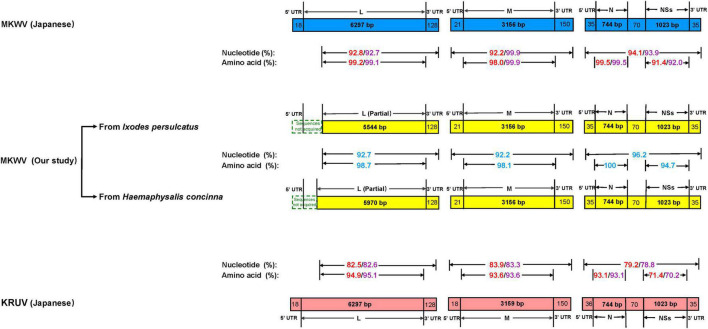
Comparison of homology between MKWV and KURV. Nucleotide and amino acid identities between Japanese MKWV, KURV, and our MKWV from *I. persulcatus* are marked with red. Nucleotide and amino acid identities’ sequences between Japanese MKWV, KURV, and our MKWV from *H. concinna* are marked with purple. The nucleotide and amino acid identities of our two MKWVs are marked with blue.

For MKWV from *I. persulcatus*, the full length of medium (M) segment (3,327 bp), small (S) segment (1,907 bp), and the nearly full length of the L segment (5,672 bp) was obtained from the cell culture (deposited in GenBank with accession numbers MZ532499-MZ532501). The pairwise similarity analysis based on 5,672 bp of partial L, 3,327 bp of full M, and 1,907 bp of full S sequences showed 92.8, 92.2, and 94.1% nucleotide acid identity between the currently sequenced MKWV and Japanese MKWV (accession numbers LC063768-LC063770) (Figure 2 and Supplementary Tables 2, 3), suggesting that this is a novel variant of MKWV. The pairwise amino acid identities between our MKWV and Japanese MKWV were 99.2% (RNA-dependent RNA polymerase), 98.0% (glycoprotein precursor), 99.5% (N), and 91.4% (NSs) (Figure 2, Supplementary Tables 2, 3 and Supplementary Figures 1–4). We further compared our MKWV with the Kuriyama virus (KURV), a close relative of MKWV discovered in 2019 (Torii et al., 2019). The pairwise nucleotide identities of our MKWV were 82.5% (5,672 bp partial L), 83.9% (full-length M), and 79.2% (full-length S) with KURV (Figure 2 and Supplementary Tables 2, 3). The pairwise amino acid identities of our MKWV were 94.9% (RNA-dependent RNA polymerase), 93.6% (glycoprotein precursor), 93.1% (N), and 71.4% (NSs) with KURV (Figure 2, Supplementary Tables 2, 3 and Supplementary Figures 1–4).

For MKWV from *H. concinna*, the full length of medium (M) segment (3,327 bp), small (S) segment (1,907 bp), and the nearly full length of the L segment (6,098 bp) were obtained (deposited in GenBank with accession numbers OM066888-OM066890). The pairwise similarity analysis based on 6,098 bp of partial L, 3,327 bp of full M, 1,907 bp of full S sequences showed 92.7, 99.9%, and 93.9% nucleotide acid identity between our MKWV and Japanese MKWV (accession numbers LC063768-LC063770) (Figure 2 and Supplementary Tables 2, 3). The pairwise amino acid identities between our MKWV and Japanese MKWV were 99.1% (RNA-dependent RNA polymerase), 99.9% (glycoprotein precursor), 99.5% (N), and 92% (NSs) (Figure 2, Supplementary Tables 2, 3 and Supplementary Figures 1–4). The pairwise nucleotide identities were 82.6% (6098 bp partial L), 83.3% (full length M), and 78.8% (full length S) between our MKWV and KURV (Figure 2 and Supplementary Tables 2, 3). The pairwise amino acid identities of our MKWV were 95.1% (RNA-dependent RNA polymerase), 93.6% (glycoprotein precursor), 93.1% (N), and 70.2% (NSs) with KURV (Figure 2, Supplementary Tables 2, 3 and Supplementary Figures 1–4).

The pairwise nucleotide identities of the MKWV from *I. persulcatus* and *H. concinna* were 92.7% (6,098 bp partial L), 92.2% (full-length M), and 96.2% (full- length S), and the pairwise amino acid identities were 98.7% (RNA-dependent RNA polymerase), 98.1% (glycoprotein precursor), 100% (N), and 94.7% (NSs) (Figure 2, Supplementary Tables 2, 3 and Supplementary Figures 1–4), suggesting that the MKWV from *H. concinna* is a novel MKWV variant.

### Phylogenetic Analysis of Mukawa Virus Sequences

**FIGURE 3:**
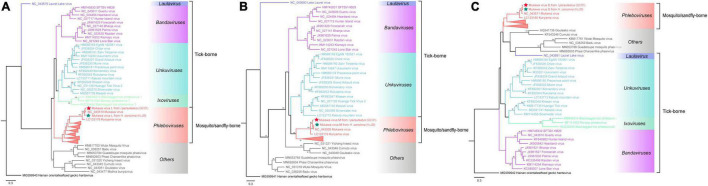
Phylogenetic analysis of MKWV RNA segments. **(A)** The ML phylogenetic tree constructed based on a 5,672-bp fragment of the partial L segment. **(B)** The ML phylogenetic tree constructed based on the full-length nucleotide sequences of the M segment. **(C)** The ML phylogenetic tree constructed based on the full-length nucleotide sequences of the S segment. Our MKWV from *I. persulcatus* was labeled with a red solid five-pointed star, and our MKWV from *H. concinna* was labeled with a green solid five-pointed star. Trees that were generated using MEGA-X and analyzed included 1,000 bootstrap replicates.

**FIGURE 4:**
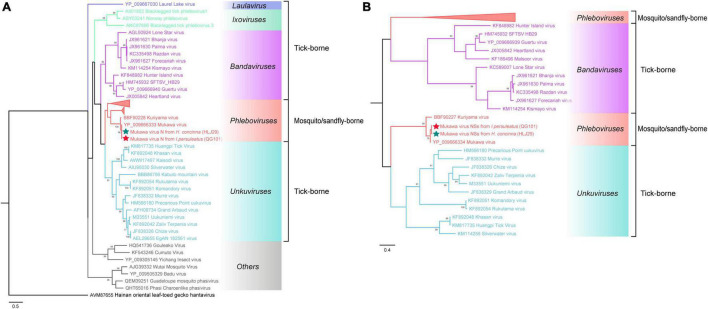
Phylogenetic analysis of MKWV N and NSs proteins. **(A)** The maximum likelihood phylogenetic tree based on the deduced amino acid sequences of the MKWV nucleoprotein protein (N). **(B)** The maximum likelihood phylogenetic tree based on the deduced amino acid sequences of the MKWV non-structural protein (NSs). Our MKWV from *I. persulcatus* was labeled with a red solid five-pointed star, and our MKWV from *H. concinna* was labeled with a green solid five-pointed star. Trees that were generated using MEGA-X and analyzed included 1,000 bootstrap replicates.

**FIGURE 5:**
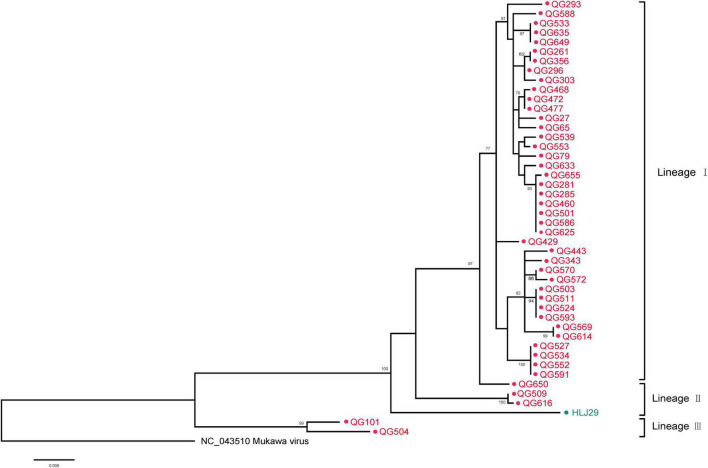
Phylogenetic analysis of 895-bp MKWV-positive RNA sequences. The Maximum likelihood phylogenetic tree constructed based on the 895-bp MKWV-positive RNA sequences (*n* = 46) of the L segment. The MKWV-positive RNA sequences from *I. persulcatus* were labeled with red solid dots, and the MKWV-positive RNA sequences from *H. concinna* were labeled with green solid dots. Trees that were generated using MEGA-X and analyzed included 1,000 bootstrap replicates.

The phylogenetic tree was constructed based on the nucleotide sequences of L, M, and S segments, demonstrating the Japanese MKWV, our MKWV, and KURV clustered with the members of the mosquito/sandfly-borne phleboviruses and distant from other tick-borne phenuiviruses (Figures 3A–C). The phylogenetic trees based on the deduced amino acid sequences of the MKWV N protein demonstrated high similarity between MKWV and mosquito/sandfly-borne phleboviruses (Figure 4A); in contrast, the phylogenetic trees based on NSs protein demonstrated that MKWV was more closely related to other tick-borne phenuiviruses (Figure 4B). Phylogenetic analysis based on 895 bp partial L gene sequences (*n* = 46) showed that our MKWV sequences were separated into three lineages, all of which were independent of the lineage of Japanese MKWV (Figure 5).

## Discussion

In this study, we confirmed the existence of MKWV among tick vectors in China with a positive rate of 2.5%, while no positive detection was observed among wild small mammals. In agreement with the discovery of MKWV in *I. persulcatus* in Japan, MKWV was also determined in *I. persulcatus* in China. *H. concinna* was also identified to carry MKWV in the current study, thus expanding the currently known range of tick species parasitized by MKWV. In addition to the successful isolation of MKWV from Huh7 and the inoculation in mice in Japan, we isolated MKWV using Vero cells, which indicated their possible pathogenicity in mammalian cells and the potential of causing human infection.

The genus *Phlebovirus* belonging to the family *Bunyaviridae* contains human pathogens that can be carried by a wide range of arthropod vectors (e.g., phlebotomine sandflies, mosquitoes, and ticks) and can infect a wide range of animals. Four TBPV groups that are genetically distinct had been traditionally classified: the SFTSV/HRTV group associated with viral hemorrhagic fever-like illness, Bhanja group associated with sporadic febrile illness, Uukuniemi and Kaisodi groups which have not been recognized as human pathogens (Matsuno et al., 2018; Shi et al., 2018). The most recent classification as of July 2019, however, had taxonomically reclassified the SFTSV/HRTV and Bhanja groups into the genus *Bandavirus*, while the Uukuniemi and Kaisodi groups were assigned to the genus *Uukuvirus* (Abudurexiti et al., 2019). There is also a stunning increase in the number of newly identified phleboviruses (e.g., Malsoor virus, Fermo virus, and Drain virus) that remain to be classified in the past decade (Mourya et al., 2014; Remoli et al., 2014; Bino et al., 2019). With the increased identification of novel viruses and the reclassifications of related virus, genus *Phlebovirus* had currently expanded to contain 66 species. The MKWV was expanded as one species in genus *Phlebovirus* in 2018 (Abudurexiti et al., 2019).

Tick-borne phenuiviruses were known to infect a variety of tick vectors. *H. Longicornis*, as one of the most widely distributed ticks, is the main tick vector of SFTSV and Khasan virus (Zhao et al., 2020). *Amblyomma Americanum*, vector of HRTV and Lone Star virus, is mainly distributed in United States and transmit more than a third of human TBD agents in United States (Raghavan et al., 2019). *Ixodes ricinus*, the most common hard tick species in Europe act as an important vector of Uukuniemi virus (Medlock et al., 2013; Tonk et al., 2014). *I. persulcatus* is one of the most widely distributed tick species in the world, with the known boundary expanded to new endemic regions, for example, its newly identification in European countries in southern Karelia (Russia) and Sweden (Cao et al., 2003; Tokarevich et al., 2011; Bugmyrin et al., 2013; Zhang et al., 2014; Jaenson et al., 2016; Wikel, 2018; Kjær et al., 2019; Madison-Antenucci et al., 2020). *H. concinna* is a widely distributed species in the temperate climate zone of Eurasia, within the belt of 28–64°N latitude, from the Spanish Atlantic coast to Kamchatka, Russia (Rubel et al., 2018; Kiewra et al., 2019; Paulauskas et al., 2020). The current findings thus highlighted the potential role of these two tick species as vectors of transmitting MKWV.

MKWV has a unique evolutionary position in phleboviruses. It is the first TBPV identified to be genetically similar to mosquito/sandfly-borne phleboviruses and the sole tick-borne member of the genus *Phlebovirus* according to the latest classification of ICTV (Matsuno et al., 2018; Abudurexiti et al., 2019). According to Matsuno et al. (2018) study, MKWV is genetically distinct from other known TBPVs and shares most similar RNA genome sequences with mosquito/sandfly-borne phleboviruses. Our study also showed similar results, indicating that the MKWV N protein branch fell into mosquito/sandfly-borne phleboviruses, while the MKWV NSs protein showed a closer relationship with other tick-borne phenuiviruses, located between genera *Uukuvirus* and *Bandavirus*. These findings might expand our understanding of the evolutionary relationship between TBVs and mosquito/sandfly-borne viruses in *Phenuivirus* family.

The glycoprotein encoded by the M fragment and the N protein encoded by the S fragment of phleboviruses have strong antigenicity and immunogenicity (Wu et al., 2014, 2017). Torii et al. (2019) have compared the serological reactivity between KURV and MKWV and found a cross-reactivity of antisera against MKWV to KURV. Based on the pairwise similarity analysis, we demonstrated high homology between Japanese MKWV, current MKWV, and KURV on glycoprotein and N protein. The serological cross-reactivity of anti-MKWV antisera with other TBPVs, especially KURV, may require further study.

In addition to the human-derived Huh-7 cell and newborn mice from which MKWV was successfully isolated or infected, we have isolated MKWV from another cell line (Vero). The infection of MKWV in mammals used to be suggested by serological tests in wildlife and the PCR detection of viral RNA in animal experiments (Torii et al., 2019). The current findings on the isolation of MKWV from mammalian cell lines thus extended the knowledge on its potential importance in causing human disease.

In summary, we found the presence of MKWV in *I. persulcatus* and *H. concinna* in China. Phylogenetic and genomic evidence supported them to be a novel variant of MKWV. These findings extend the current knowledge on the known tick vectors that had previously been characterized for MKWV. A wide screen for MKWV is needed in regions where these two tick species are highly abundant. The potential risks to humans should be performed by serological study. Research into different MKWV variants and related viruses like KURV is also warranted to help increase our understanding of the molecular evolution and mechanisms of their pathogenesis.

## Data Availability Statement

Th datasets presented in this study can be found in online repositories. The names of the repository/repositories and accession number(s) can be found below: https://www.ncbi.nlm.nih.gov/genbank/, MZ532499, https://www.ncbi.nlm.nih.gov/genbank/, MZ532500, https://www.ncbi.nlm.nih.gov/genbank/, MZ532501, https://www.ncbi.nlm.nih.gov/genbank/, OM066888, https://www.ncbi.nlm.nih.gov/genbank/, OM066889, https://www.ncbi.nlm.nih.gov/genbank/, OM066890, https://www.ncbi.nlm.nih.gov/genbank/, OL439499-OL439542.

## Ethics Statement

The animal study was reviewed and approved by the Institute of the Academy of Military Medical Sciences.

## Author Contributions

WL and X-AZ: conceptualization. Y-NW, R-RJ, HD, NW, Y-FZ, YL, J-JC, and GW: experiment performers. Y-NW: writing – original draft preparation. Y-NW, R-RJ, X-LZ, P-HZ, J-FJ, L-ZL, HL, M-bY, X-AZ, and WL: writing – review and editing. WL and X-AZ: supervision. All authors have read and agreed to the published version of the manuscript.

## Conflict of Interest

The authors declare that the research was conducted in the absence of any commercial or financial relationships that could be construed as a potential conflict of interest.

## Publisher’s Note

All claims expressed in this article are solely those of the authors and do not necessarily represent those of their affiliated organizations, or those of the publisher, the editors and the reviewers. Any product that may be evaluated in this article, or claim that may be made by its manufacturer, is not guaranteed or endorsed by the publisher.
