# Supplementary material for: First Detection of Mukawa Virus in Ixodes persulcatus and Haemaphysalis concinna in China
Source: Front Microbiol. 2022 Mar 3;13:791563. doi: 10.3389/fmicb.2022.791563 (PMC8930188; doi:10.3389/fmicb.2022.791563)
Supplement: Supplementary file 1 [file Data_Sheet_1.docx]

**Figure S1. Amino acid alignment of the L protein Japanese MKWV, our MKWV and KURV.** Amino acid alignment was performed using ESPript 3.0 (<http://espript.ibcp.fr/ESPript/cgi-bin/ESPript.cgi>).


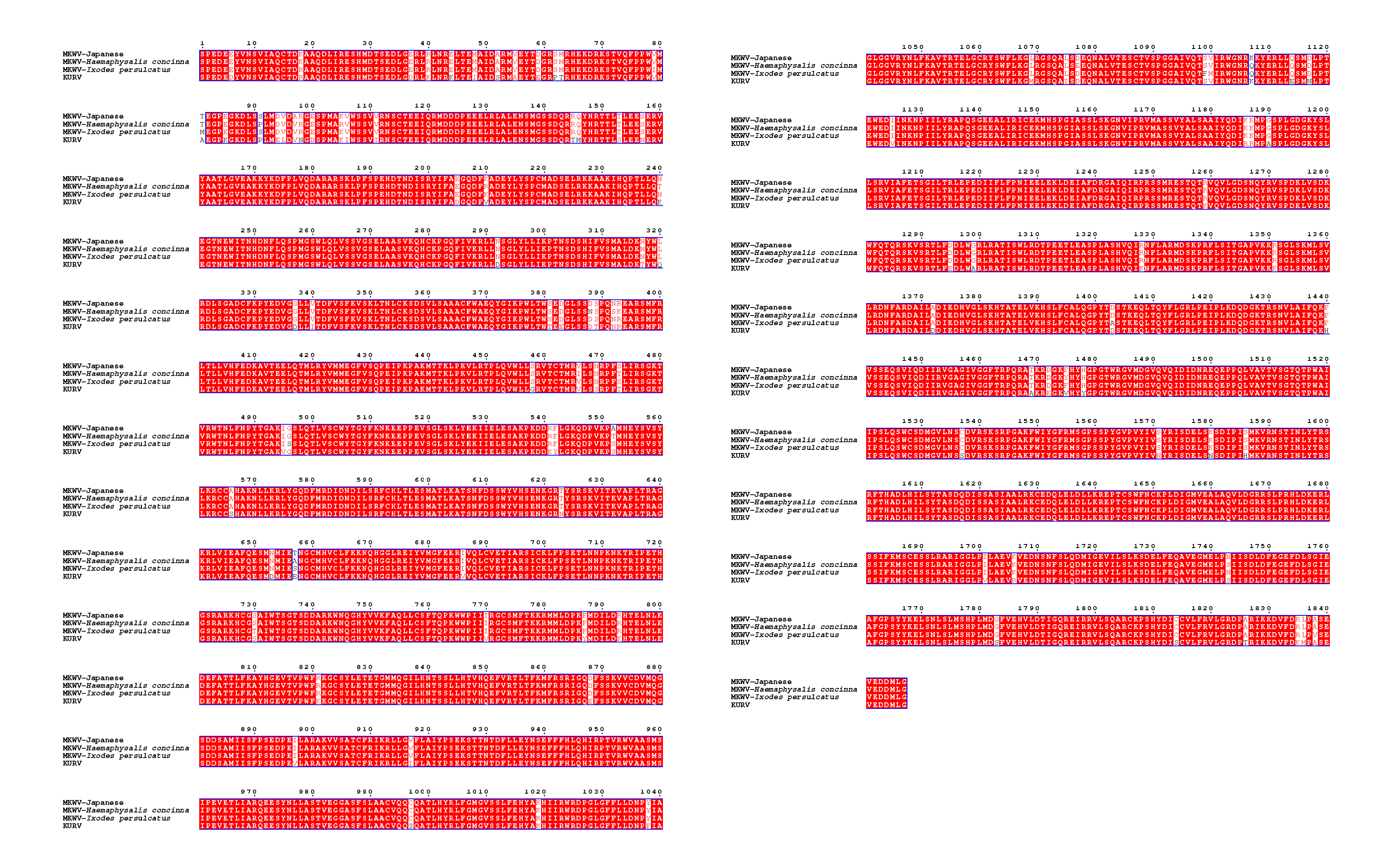


**Figure S2. Amino acid alignment of the glycoprotein precursor between Japanese MKWV, our MKWV and KURV.**


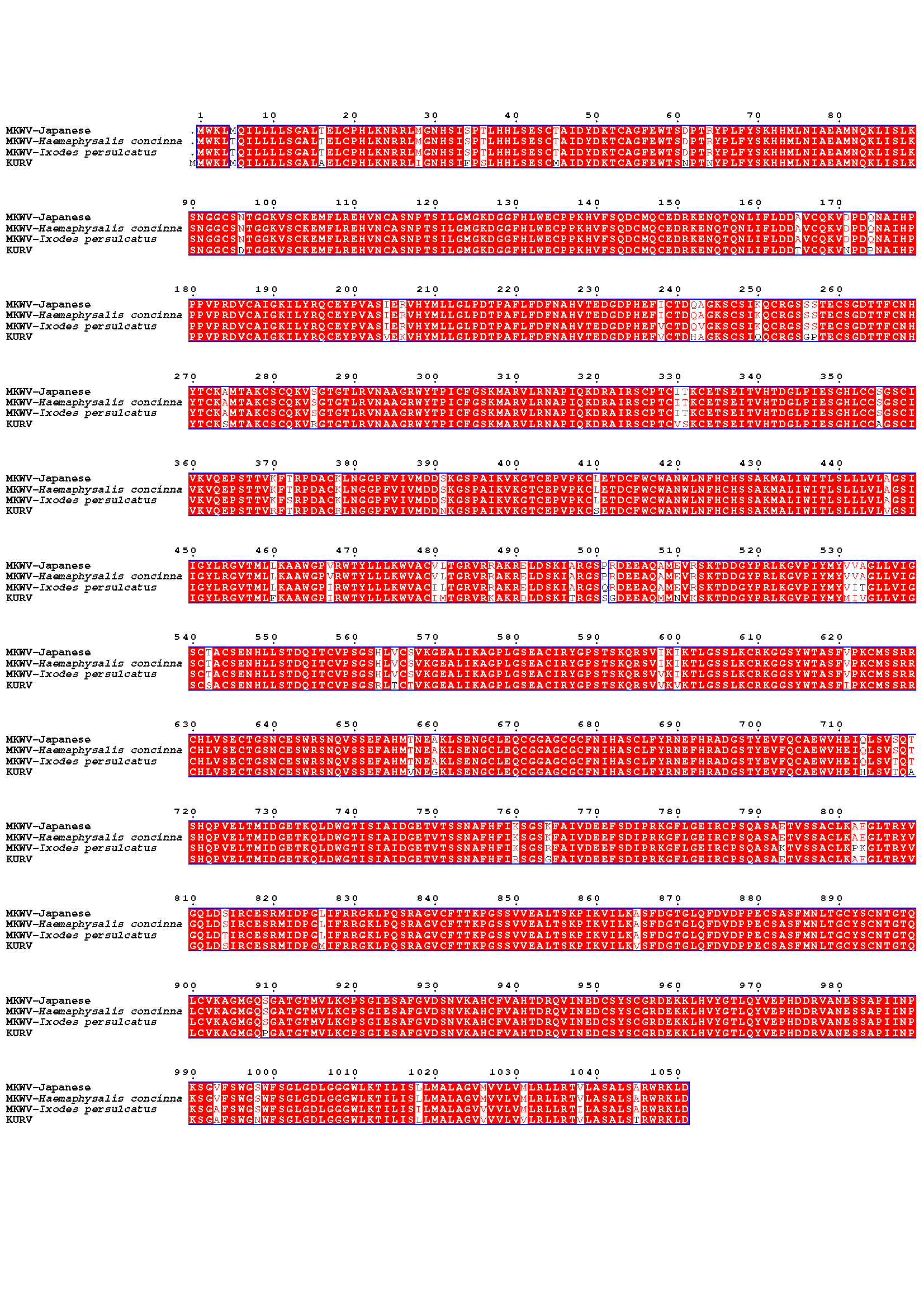


**Figure S3. Amino acid alignment of the N protein Japanese between Japanese MKWV, our MKWV and KURV.**


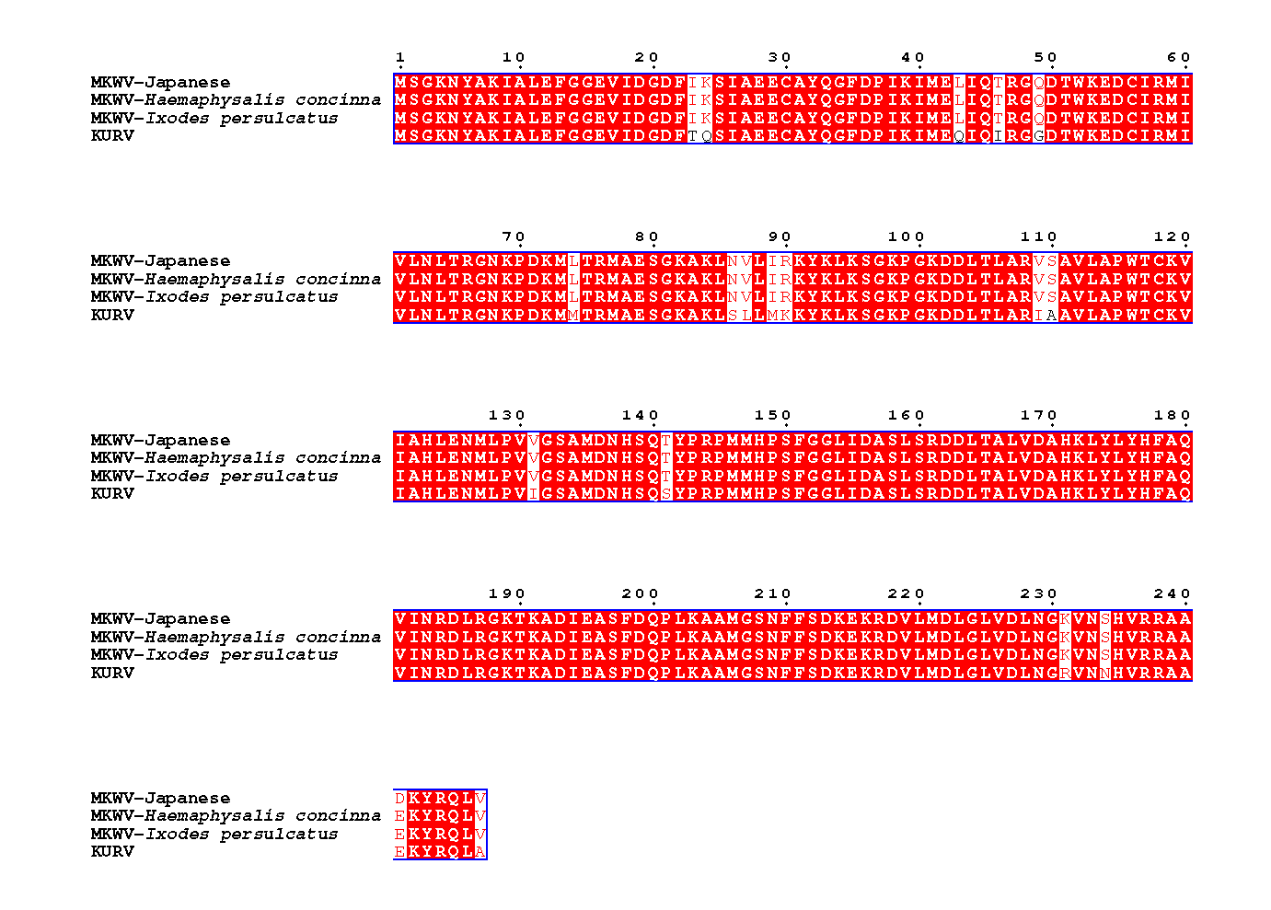


**Figure S4. Amino acid alignment of the NSs protein between Japanese MKWV, our MKWV and KURV.**


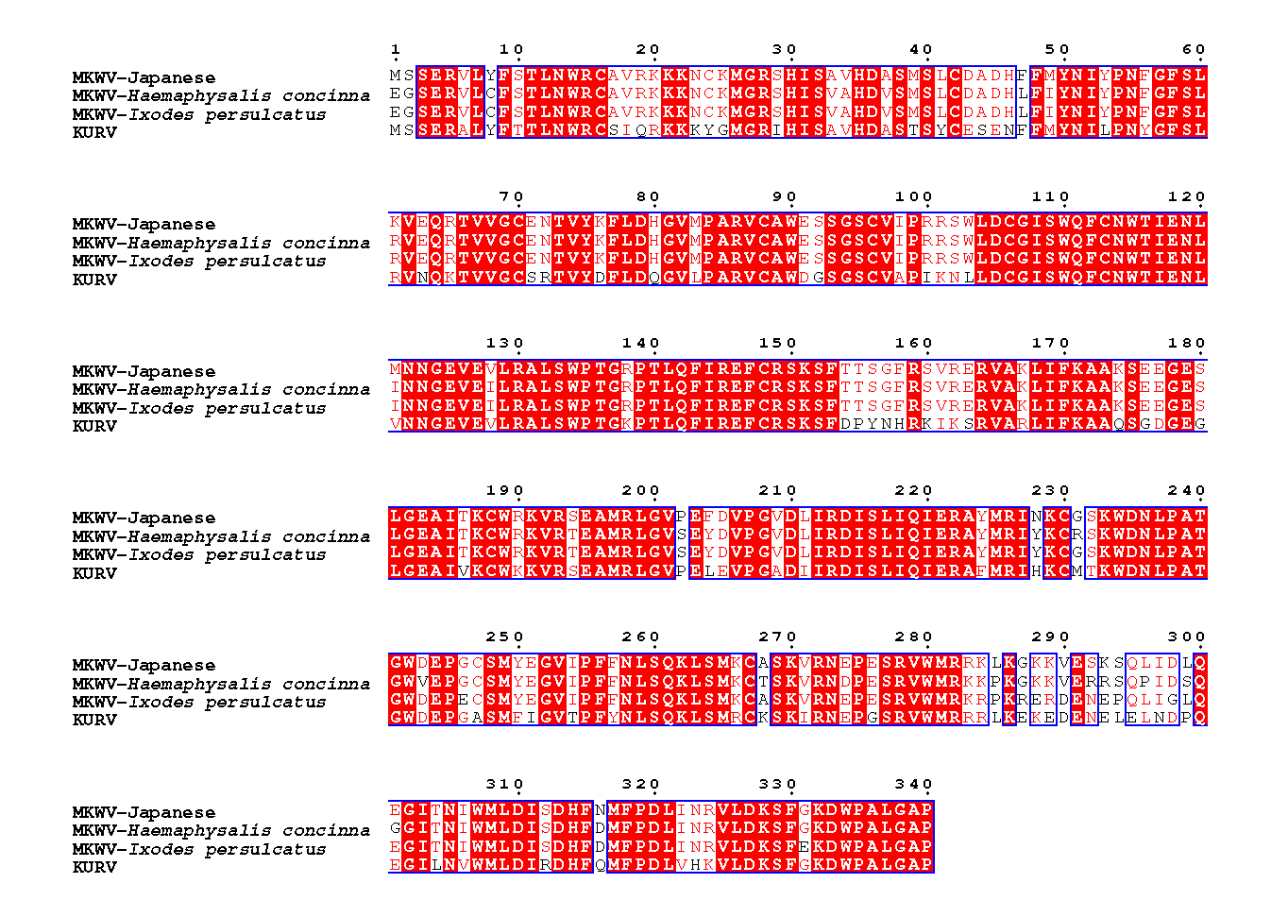


**Table S1. Mukawa virusspecific primer pairs for the genome sequencing.**

| **ID** | **Primer name** | **Forward primer** | **Reverse primer** | **Product size(bp)** | **Gene regions** |
| --- | --- | --- | --- | --- | --- |
| 1 | L-772-1737 | TCCCCAGAAGATGAAGAGTACG | ATCTCTGAGCCAGTAGTTCT | 964 | L |
| 2 | L-1620-2458 | CCAGTTCATTGTTAAGAGGC | TCTTCAGGTAGGACACACTAT | 839 | L |
| 3 | L-2389-2826 | GATAGATTCCTGGGAAAGCA | CCTCTCCTCAAATCCCATCA | 438 | L |
| 4 | L-2779-3287 | CAGCATGGIGGICTIAGAGAGAT | TGIAGIATSCCYTGCATCAT | 509 | L |
| 5 | L-3217-4029 | ACAGTTCCATGGTTTAGAAAG | GCTCTCAGTGACCAATGC | 813 | L |
| 6 | L-3882-4168 | ATACATTGCAGGCCTTGGAG | GGATGATCGGGTTCTTGTTG | 287 | L |
| 7 | L-4058-4989 | TTCAGACATCAGTGATCAGG | CTTTGTCGAGTCCGTGTATG | 932 | L |
| 8 | L-4932-5780 | GCTTGTGAAGCACTCTCTTT | GATCTCCTCCCATCTAGGAC | 849 | L |
| 9 | L-5549-6443* | CCATCAATCTGTACACCAGG | ACACAAAGTCCGCCCATTAC | 895 | L |
| 10 | M-1-684 | ACACAAAGACGGCGCTTCATT | TAGAAAGGCTGGGGTGTCTG | 684 | M |
| 11 | M-612-1368 | TGAATACCCTGTCGCTAGTA | TATGGATCCAGCAAGGACTA | 757 | M |
| 12 | M-997-1975 | TGTCCAACATGCATCACTAA | CAGAGCTCACCTGATTAGAC | 979 | M |
| 13 | M-1698-2441 | ATCTGGAAGCCATCTTGTTT | CTTGTTAGTCCCTCTGCTTT | 744 | M |
| 14 | M-2157-2565 | TCAATTGAGTGTGTCCCAAA | GGAACCTGGCTTTGTAGTAA | 409 | M |
| 15 | M-2421-2862 | GAAAGCAGAGGGACTAACAA | TATGACCTGCCTATCGGTAT | 442 | M |
| 16 | M-2719-3327 | TTGTGTGTGAAAGCTGGCAT | ACACAAAGACCGGCGCAAAT | 609 | M |
| 17 | S-1-324 | ACACAAAGACCCCTCTCTCG | CGCTCTTGAGCTTGTACTTC | 324 | S |
| 18 | S-163-990 | TCATTCAAACCAGAGGTCAG | CAGCTCATCGACTTACAAGA | 828 | S |
| 19 | S-971-1381 | TCTTGTAAGTCGATGAGCTG | GGTGGCTAAGCTCATATTCA | 411 | S |
| 20 | S-1235-1875 | CTTATCAGATCGACACCAGG | AAGATGAGTTCTGAACGTGT | 641 | S |
| 21 | S-1537-1907 | AAACTGCCACGAGATTCC | ACACAAAGACCCCCTTTCAT | 371 | S |

* The primer ID9 is the primer setL-5549-6443 in Table 1.

**Table S2.**Nucleotide similarity (%) between MKWV and KURV*.

| **Nucleotide** | **Virus** | **MKWV**  **(From *I. persulcatus*)** | **MKWV**  **(From *H. concinna*)** | **MKWV (Japanese)** | **KURV** |
| --- | --- | --- | --- | --- | --- |
| L |  |  |  |  |  |
|  | MKWV (From *I. persulcatus*) | *** | 92.7 | 92.8 | 82.5 |
|  | MKWV (From *H. concinna*) | *** | *** | 92.7 | 82.6 |
|  | MKWV (Japanese) | *** | *** | *** | 82.5 |
|  | KURV | *** | *** | *** | *** |
| M |  |  |  |  |  |
|  | MKWV (From *I. persulcatus*) | *** | 92.2 | 92.2 | 83.9 |
|  | MKWV (From *H. concinna*) | *** | *** | 99.9 | 83.3 |
|  | MKWV (Japanese) | *** | *** | *** | 83.4 |
|  | KURV | *** | *** | *** | *** |
| S |  |  |  |  |  |
|  | MKWV (From *I. persulcatus*) | *** | 96.2 | 94.1 | 79.2 |
|  | MKWV (From *H. concinna*) | *** | *** | 93.9 | 78.8 |
|  | MKWV (Japanese) | *** | *** | *** | 80.1 |
|  | KURV | *** | *** | *** | *** |

* MKWV, Mukawa virus; KURV, Kuriyama virus;*I. persulcatus, Ixodes persulcatus; H. concinna,Haemaphysalisconcinna.*

**Table S3.**Amino acid similarity (%) between MKWV and KURV*.

| **Amino acid** | **Virus** | **MKWV**  **(From *I. persulcatus*)** | **MKWV**  **(From *H. concinna*)** | **MKWV**  **(Japanese)** | **KURV** |
| --- | --- | --- | --- | --- | --- |
| RdRp |  |  |  |  |  |
|  | MKWV (From *I. persulcatus*) | *** | 98.7 | 99.2 | 95.0 |
|  | MKWV (From *H. concinna*) | *** | *** | 99.1 | 95.1 |
|  | Japanese MKWV | *** | *** | *** | 95.2 |
|  | KURV | *** | *** | *** | *** |
| Glycoprotein |  |  |  |  |  |
|  | MKWV (From *I. persulcatus*) | *** | 98.1 | 98.0 | 93.6 |
|  | MKWV (From *H. concinna*) | *** | *** | 99.9 | 93.6 |
|  | Japanese MKWV | *** | *** | *** | 93.7 |
|  | KURV | *** | *** | *** | *** |
| N |  |  |  |  |  |
|  | MKWV (From *I. persulcatus*) | *** | 100.0 | 99.5 | 93.1 |
|  | MKWV (From *H. concinna*) | *** | *** | 99.5 | 93.1 |
|  | Japanese MKWV | *** | *** | *** | 92.7 |
|  | KURV | *** | *** | *** | *** |
| NSs |  |  |  |  |  |
|  | MKWV (From *I. persulcatus*) | *** | 94.7 | 91.4 | 71.4 |
|  | MKWV (From *H. concinna*) | *** | *** | 92.0 | 70.2 |
|  | Japanese MKWV | *** | *** | *** | 75.0 |
|  | KURV | *** | *** | *** | *** |

* RdRp, RNA-dependent RNA polymerase; N, nucleocapsid protein; NSs, nonstructural protein; MKWV, Mukawa virus; KURV, Kuriyama virus; *I. persulcatus, Ixodes persulcatus; H. concinna,Haemaphysalisconcinna.*
